# A comparative approach to quantifying provision of acute therapy services

**DOI:** 10.1097/MD.0000000000027377

**Published:** 2021-10-08

**Authors:** Carmen E. Capo-Lugo, Robert L. Askew, Matthew Boebel, Christine DeLeo, Anne Deutsch, Allen Heinemann

**Affiliations:** a Department of Physical Therapy, School of Health Professions, University of Alabama at Birmingham, AL; b Department of Psychology, Stetson University, DeLand, FL; c Shirley Ryan AbilityLab, Chicago, IL; d Center for Rehabilitation Outcomes Research, Shirley Ryan AbilityLab, Chicago, IL; e Department of Physical Medicine and Rehabilitation, Feinberg School of Medicine, Northwestern University, Chicago, IL; f RTI International, Chicago, IL.

**Keywords:** health services research, logistic models, process assessment (health care), quality improvement, rehabilitation

## Abstract

This study aims to compare delivery of acute rehabilitation therapy using metrics reflecting distinct aspects of rehabilitation therapy services. Seven general medical-surgical hospitals in Illinois and Indiana prospectively collected rehabilitation therapy data. De-identified data on all patients who received any type of acute rehabilitation therapy (n = 35,449) were extracted and reported as aggregate of minutes of therapy services per discipline. Metrics included therapy types, total minutes, and minutes per day (intensity), as charted by therapists. Extended hospital stay was defined as a length of stay (LOS) longer than Medicare's geometric mean LOS. Discharge destination was coded as postacute care or home discharge. Substantial variability was observed in types, number of minutes, and intensity of therapy services by condition and hospital. The odds of an extended hospital stay increased with increased number of minutes, increased number of therapy types, and decreased with increased rehabilitation intensity. This comparative approach to assessing provision of acute therapy services reflect differential effects of service provision on LOS and discharge destination. Investigators, policymakers, and hospital administrators should examine multiple metrics of rehabilitation therapy provision when evaluating the impact of health care processes on patient outcomes.

## 1 Introduction

Delivery of rehabilitation therapy (i.e., physical, occupational, and speech and language) services during acute care hospitalizations is associated with reductions of in-hospital functional decline, shorter recovery times, fewer readmissions, cost savings, and global improvements in health.^[1–6]^ However, the type and amount of therapy services provided to patients in acute care varies widely.^[7,8]^ The lack of clear evidenced-based practice guidelines and institutional pressures to minimize costs compound variability.^[7]^ Although not all variability is undesirable, increased variability in healthcare utilization is associated with increased costs and lower quality care.^[13]^

Acute care therapy delivery varies in terms of documentation, prioritization, total number of minutes, and intensity patterns.^[4,9–12]^ Studies vary widely in how they define and report utilization and delivery of therapy services. For example, Freburger et al^[10–12]^ examined factors associated with delivery of physical therapy (PT) and defined intensity using revenue codes (i.e., total PT codes/total revenue codes). Similarly, Rogers et al^[6]^ defined intensity of services using Medicare claims data to assess the association between delivery of in-hospital services and overall hospital spending. Jette et al^[9]^ used therapists’ documentation to create multiple metrics of therapy provision (e.g., total number of visits, average duration). These varied methodologies reflect distinct aspects of acute care therapy service delivery or utilization; however, their varying definitions and metrics across studies limits our understanding of the relationships between therapy services and patient outcomes. Further, use of data from only 1 discipline provides limited information regarding the effectiveness of acute therapy because therapy services are typically coordinated across therapy disciplines (e.g., within specialty units like stroke units).

The objectives of this study were to describe and compare variability in therapy service using metrics reflecting distinct aspects of therapy services, and to explore how variations in therapy definitions and metrics are associated with hospital length of stay (LOS) and discharge destination. These findings can serve as baseline reference points for future comparisons of practice patterns and process outcomes as care move towards advanced payment models.^[14]^ Moreover, findings will facilitate more nuanced characterizations of acute therapy service delivery and their relationship to important process outcomes.

## 2 Methods

Data are from 7 short-term acute care hospitals in Illinois and Indiana shared through a quality improvement initiative in 2012. The Institutional Review Board at our institution deemed this project as quality improvement and required no further review. All patients who received any type of therapy service while hospitalized were included. Patients did not provide written informed consent, as our study was focused on examining patterns of therapy provision and did not affect the care patients received. Hospitals extracted de-identified data and reported an aggregate of minutes of therapy services per discipline.

LOS represents number of days between hospital admission and discharge. Extended hospital stays were defined as LOS greater than Medicare's geometric mean LOS for each Medicare Severity-Diagnostic Related Group (MS-DRG) code (i.e., patient LOS > Medicare's geometric mean LOS). Discharge destination is a binary variable representing discharge to postacute care (PAC; hospice, long-term care facility, inpatient rehabilitation facility, or skilled nursing facility vs home discharge).

Total number of minutes of acute therapy services was defined as total minutes of PT, occupational therapy (OT), and speech and language pathology. Distinct therapy types were defined as the number of acute therapy services (i.e., PT, OT, and/or speech and language pathology) provided to patients. Therapy intensity was defined as the average minutes of therapy services per day.

We removed MS-DRG 945 and 946 (“Rehabilitation”) because these categories are heterogeneous, describing treatment rather than a medical condition or procedure.^[12]^ Next, we created diagnostic groups for the most frequently observed MS-DRG codes (i.e., stroke [excluding transient ischemic attacks]: 61–68; chronic obstructive pulmonary disease [COPD]: 190, 191, 192; pneumonia: 193, 194, 195; heart failure [HF]: 291, 292, 293; major joint replacement [MJR]: 469, 470; and septicemia: 870, 871, 872). Within each diagnostic group, MS-DRG codes indicated absence or presence of minor or major comorbidities or complications, which we used as a proxy indicator of severity and comorbidities.

To assess the effects of acute therapy services on the odds of an extended hospital stay, we employed a mixed effects logistic regression model for each diagnostic group, which included fixed effects of service provision (total number of minutes, distinct therapy types, and therapy intensity) and random effects for hospitals. Fixed effects were stratified by total number of minutes (<120, 120–239, ≥240), distinct therapy types (1, 2, or 3), and therapy intensity (<30, 30–59, ≥60 minutes per day). Odds ratios (ORs) and 95% confidence intervals were estimated controlling for hospital size and condition severity/comorbidities. We excluded outliers, that is the top 1% (n = 104) in each diagnostic category to mitigate leverage on estimates (i.e., MJR ≥ 170 minutes/day; HF ≥ 71 minutes/day; COPD ≥ 75 minutes/day; pneumonia ≥ 84 minutes/day; septicemia ≥ 82.5 minutes/day; stroke ≥ 165 minutes/day). Likelihood ratio *X*^2^ tests guided comparisons of absolute and relative model fit when constructing the mixed effects models. To assess the effects of service provision on PAC discharge, we employed the same statistical approach detailed above. ORs and 95% confidence intervals controlled for hospital size, condition severity, age, and payor. Statistical analyses were conducted with STATA/IC 12.1 for Mac (StataCorp LLC, College Station, TX).

## 3 Results

### 3.1 Hospital, patients, and therapy characteristics

Table 1 shows the 35,449 patients (mean age = 69.0; 70.2% Medicare) received rehabilitation therapy (minutes of therapy interquartile range [IQR] = 60–225; minutes per day IQR = 15–49). Among the 6 hospital that provided therapy data stratified by type of service, PT (minutes per day IQR = 12–36) was most common. By design, all patients received at least 1 form of therapy; approximately 66% received PT along with OT, and/or speech and language therapy. The most commonly observed MS-DRGs were MJR (9.5%) followed by septicemia (4.3%), stroke (3.8%), HF (3.6%), pneumonia (3.3%), and COPD (2.7%).

**Table 1:** Hospital and patient characteristics.

		Hospitals						
	Entire sample (n = 35449)	#1^∗^ (n = 9973)	#2 (n = 6377)	#3 (n = 5423)	#4 (n = 4792)	#5 (n = 3465)	#6 (n = 3117)	#7 (n = 2302)
**Hospital beds**, n	2284	600	387	339	296	408	140	114
**Geographic region**	–	IN-other	IL-large	IL-large	IL-large	IL-large	IL- other	IL- other
**Hospital type**	–	Non-academic	Non-academic	Non-academic	Non-academic	Academic	Academic	Non-academic
**Length of stay**, days, median ± IQR	5 [3–8]	6 [4–11]	5 [3–8]	4 [3–7]	4 [3–7]	4 [3–8]	3 [2–6]	5 [3–9]
**Age,** mean ± SD	69.0 ± 17.8	71.2 ± 16.7	70.4 ± 15.9	67.8 ± 19.8	69.4 ± 17.3	61.3 ± 22.0	68.1 ± 16.1	71.6 ± 14.5
**Health insurance**, %								
Medicare	70.2	76.2	69.3	64.4	68.8	61.0	68.4	79.5
Private	19.1	14.1	22.7	22.9	23.6	25.1	15.1	8.8
Other (Medicaid, uninsured)	10.7	9.7	8.0	12.7	7.6	13.9	16.5	11.7
**Discharge destination**, %								
Home	58.7	57.9	57.6	57.1	63.3	53.0	63.3	61.8
Postacute (IRF, SNF, Hospice)	39.7	39.6	41.8	41.2	35.3	45.5	34.5	37.2
Other (expired, left against medical advice)	1.7	2.5	0.6	1.7	1.4	1.6	2.3	1.0
**Diagnostic subgroups (DRGs)**, %	27.3	26.7	28.2	29.1	24.8	19.2	33.8	31.0
Major joint replacement (DRGs 469–470)	9.5	7.3	11.5	7.9	8.5	9.2	15.2	12.4
Septicemia (DRGs 870–872)	4.3	3.7	4.0	7.8	2.1	1.6	6.0	5.3
Stroke (DRGs 61–68)	3.8	4.1	4.5	3.2	4.0	2.2	5.0	2.7
Heart failure (DRGs 291–293)	3.6	5.0	3.1	3.2	4.4	2.4	2.5	1.8
Pneumonia (DRGs 193–195)	3.3	3.3	2.8	4.1	3.3	2.0	3.1	5.3
Chronic obstructive pulmonary disease (DRGs 190–192)	2.7	3.3	2.3	2.9	2.6	1.9	2.1	3.6
**Rehabilitation therapy services**								
Total therapy minutes, median ± IQR	120 [60–225]	120 [60–240]	150 [90–255]	195 [105–315]	90 [60–180]	75 [45–135]	90 [45–150]	150 [60–690]
Therapy minutes per day, median ± IQR	25.7 [15–49]	18.6 [10–37.5]	30 [18–52.5]	40.4 [25–65]	23 [15–42]	18 [9.2–33.8]	25 [15–45]	37.5 [15–128.6]
Therapy complexity, 1 service, %	35.6^∗^	–	25.1	35.0	46.5	47.0	32.4	25.0

Diagnostic subgroups represent the combination of 2 or 3 distinct diagnostic-related group (DRG) codes. Each DRG indicates the absence or presence of minor or major comorbidities or complications. IL = Illinois, IN = Indiana, IQR = interquartile range, IRF = inpatient rehabilitation facility, SD = standard deviation, SNF = skilled nursing facility.

∗ Hospital 1 only provided total minutes of therapy.

### 3.2 Therapy service variability

**Figure 1:**
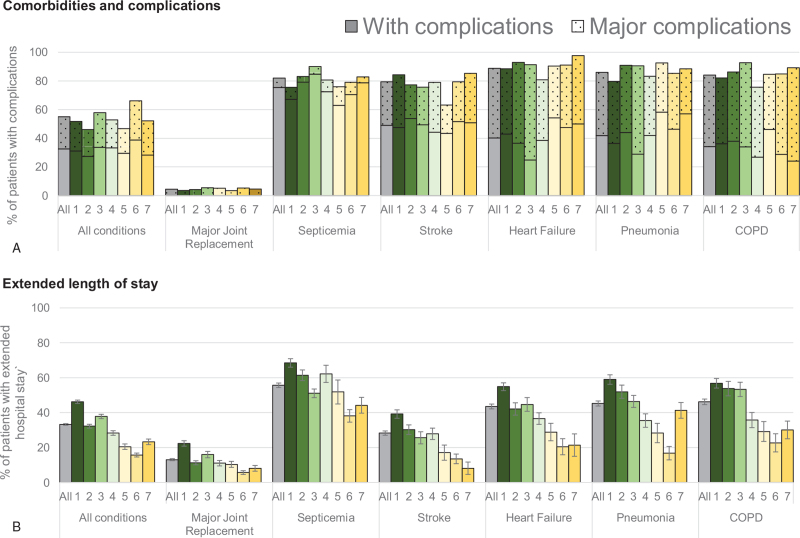
Distribution of therapy services provided to patients with (A) complications and comorbidities and (B) extended length of stay by hospital and condition. Data for each hospital are presented by condition. (A) Percent of patients who had complicating or comorbid condition (solid shading) or major complicating or comorbid condition (shading with dots) as indicated by MS-DRG) codes. (B) Percent of patients with an extended hospital length of stay by hospital and MS-DRG. The number of the hospitals represent their size from the largest (#1) to the smallest (#7) hospital. COPD = chronic obstructive pulmonary disease, MS-DRG = Medicare Severity-Diagnostic Related Group.

The majority of patients with the 6 most common conditions (n = 9661) had complications or comorbidities (32.6%) or major complications or morbidities (22.4%); this estimate varied by condition and hospital (Fig. 1A) with the fewest complications (4.5%) observed for MJR. Likewise, the percentage of patients with an extended LOS (33.3%) varied by condition and hospital, with the fewest observed for MJR (13.1%). Figure 1B shows a trend of decreased percentage of patients with an extended hospital stay as size of the hospital decreased. This effect was observed across all 6 conditions.

**Figure 2:**
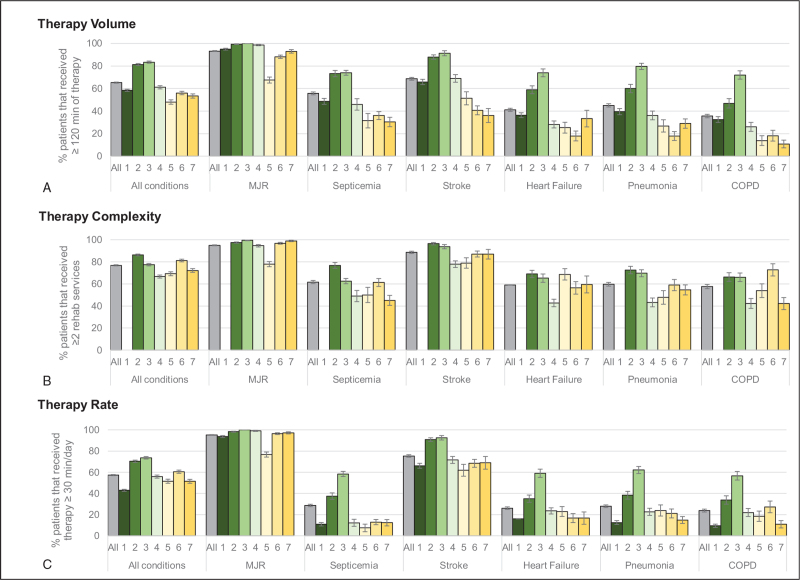
Distribution of (A) total number of minutes, (B) distinct therapy types, and (C) intensity of therapy services by hospital and condition. Data for each hospital are presented by condition. The number of the hospitals represent their size from the largest (#1) to the smallest (#7) hospital. Hospital 1 only provided total minutes of therapy. COPD = chronic obstructive pulmonary disease, MJR = major joint replacement.

While moderately strong correlations were observed among each metric of service provision (*ρ* = 0.41−0.60), Figure 2 shows substantial variability in the total number of minutes, distinct therapy types, and intensity of therapy services by condition across hospitals. Patients with MJR received the highest total number of minutes, distinct therapy types, and therapy intensity, followed by patients with stroke and septicemia. All other conditions, except MJR, showed large variability in therapy total number of minutes, distinct therapy types, and therapy intensity across hospitals.

### 3.3 Effect on extended LOS

Across conditions, higher total number of therapy minutes was associated with increased odds of an extended hospital stay, with the strongest effect observed for patients with HF (Table 2). Likewise, higher numbers of distinct service types was associated with increased odds of an extended hospital stay for all conditions except for MJR and stroke when comparing 2 vs 1 type of therapy. In contrast, increased therapy intensity was associated with decreased odds of an extended hospital stay across conditions (OR = 0.02–0.23) when comparing ≥60 vs <30 minutes of therapy per day.

**Table 2:** Effect^∗^ of volume, intensity, and complexity of therapy services on odds of (1) an extended hospital length of stay and (2) high-cost postacute care discharge.

	Major joint replacement	Septicemia	Stroke	Heart failure	Pneumonia	COPD
Odds of an extended hospital length of stay						
Total therapy minutes (volume)	OR (95% CI) n = 3335	OR (95% CI) n = 1493	OR (95% CI) n = 1336	OR (95% CI) n = 1277	OR (95% CI) n = 1153	OR (95% CI) n = 961
<120 minutes (34.78%)	REF	REF	REF	REF	REF	REF
120–239 minutes (33.01%)	1.33 (0.74–2.37)	2.87^∗∗^ (2.17–3.79)	2.52^∗∗^ (1.67–3.81)	3.72^∗∗^ (2.77–5.00)	3.14^∗∗^ (2.33–4.24)	3.36^∗∗^ (2.40–4.70)
≥240 minutes (32.21%)	3.46^∗∗^ (1.93–6.18)	19.73^∗∗^ (13.36–29.14)	12.12^∗∗^ (7.88–18.66)	45.60^∗∗^ (23.60–88.12)	19.07^∗∗^ (11.24–32.38)	37.77^∗∗^ (14.52–98.25)
Therapy types	n = 2613	n = 1125	n = 923	n = 774	n = 824	n = 632
1 service (23.14%)	REF	REF	REF	REF	REF	REF
2 services (61.69%)	1.86 (0.87–3.97)	1.95^∗∗^ (1.48–2.58)	1.57 (0.75–3.28)	1.66^∗∗^ (1.18–2.32)	1.58^∗∗^ (1.13–2.19)	1.56^∗∗^ (1.09–2.24)
3 services (15.16%)	8.12^∗∗^ (2.74–24.08)	6.23^∗∗^ (4.22–9.19)	2.53^∗∗^ (1.28–5.02)	8.62^∗∗^ (3.95–18.81)	3.68^∗∗^ (2.27–5.96)	5.76^∗∗^ (2.37–13.97)
Therapy minutes per day (intensity)	n = 3335	n = 1492	n = 1336	n = 1277	n = 1153	n = 961
<30 minutes (42.73%)	REF	REF	REF	REF	REF	REF
30–59 minutes (26.16%)	0.57^∗∗^ (0.37–0.87)	0.73^∗∗^ (0.57–0.93)	0.76 (0.55–1.03)	0.35^∗∗^ (0.26–0.48)	0.49^∗∗^ (0.35–0.67)	0.30^∗∗^ (0.20–0.44)
≥60 minutes (31.11%)	0.08^∗∗^ (0.05–0.13)	0.23^∗∗^ (0.11–0.47)	0.23^∗∗^ (0.16–0.35)	0.15^∗∗^ (0.04–0.51)	0.02^∗∗^ (0.00–0.15)	0.05^∗∗^ (0.1–0.23)
Odds of high-cost postacute care discharge						
Total therapy minutes (volume)	OR (95% CI) n = 3328	OR (95% CI) n = 1405	OR (95% CI) n = 1308	OR (95% CI) n = 1250	OR (95% CI) n = 1128	OR (95% CI) n = 956
<120 minutes (34.78%)	REF	REF	REF	REF	REF	REF
120–239 minutes (33.01%)	0.45^∗∗^ (0.31–0.64)	1.30 (0.99–1.71)	3.11^∗∗^ (2.25–4.30)	1.84^∗∗^ (1.40–2.41)	1.52^∗∗^ (1.14–2.01)	1.83^∗∗^ (1.33–2.51)
≥240 minutes (32.21%)	0.49^∗∗^ (0.34–0.71)	2.04^∗∗^ (1.50–2.77)	4.77^∗∗^ (3.30–6.88)	2.25^∗∗^ (1.53–3.29)	1.96^∗∗^ (1.31–2.92)	3.69^∗∗^ (2.28–5.98)
Therapy types	n = 2607	n = 1068	n = 906	n = 759	n = 811	n = 628
1 service (23.14%)	REF	REF	REF	REF	REF	REF
2 services (61.69%)	1.09 (0.72–1.65)	1.25 (0.93–1.67)	1.86^∗∗^ (1.05–3.29)	1.53^∗∗^ (1.11–2.10)	0.86 (0.63–1.18)	1.19 (0.82–1.72)
3 services (15.16%)	11.88^∗∗^ (2.53–55.67)	2.58^∗∗^ (1.75–3.80)	2.80^∗∗^ (1.63–4.82)	1.28 (0.65–2.51)	2.01^∗∗^ (1.26–3.21)	6.50^∗∗^ (2.67–15.84)
Therapy minutes per day (intensity)	n = 3328	n = 1405	n = 1308	n = 1250	n = 1128	n = 956
<30 minutes (42.73%)	REF	REF	REF	REF	REF	REF
30–59 minutes (26.16%)	0.68 (0.44–1.05)	1.28 (0.97–1.69)	1.59^∗∗^ (1.16–2.17)	1.22 (0.92–1.61)	1.15 (0.85–1.54)	1.47^∗∗^ (1.05–2.05)
≥60 minutes (31.11%)	0.30^∗∗^ (0.19–0.46)	2.25^∗∗^ (1.05–4.83)	1.74^∗∗^ (1.22–2.49)	1.87 (0.75–4.66)	1.06 (0.53–2.12)	0.90 (0.35–2.29)

CI = confidence interval, COPD = chronic obstructive pulmonary disease, OR = odds ratio.

∗ Estimates of effect controlling for disease severity, hospital size (number of beds), payor, and age.

∗∗ *P* < .05.

### 3.4 Effects on discharge destination

For most conditions, increased total number of minutes (i.e., ≥120 and ≥240 minutes vs <120 minutes) was associated with increased odds of PAC discharge, with the strongest effect observed for stroke, followed by COPD and HF (Table 2). Increased therapy types (3 vs 1 service) was also associated with increased odds of PAC discharge for all conditions except HF. When comparing 2 vs 1 therapy service, only stroke and HF were associated with increased odds of PAC discharge. Increased therapy intensity was associated with decreased odds of PAC discharge only for MJR (≥60 vs <30 minutes: OR = 0.30) and increased odds of PAC discharge for stroke (OR = 1.59–1.74), septicemia (≥60 vs <30 minutes: OR = 2.25), and COPD (≥30 vs <30 minutes: OR = 1.47).

## 4 Discussion

This project aims to make 3 distinct contributions to the study of acute therapy services, in that it: examines variability in acute therapy services using several therapy metrics, explores their association with acute care hospital LOS and discharge destination, and quantifies provision of acute therapy services using minutes of services as documented by therapists. With respect to describing variability in service provision, as expected, there is substantial variability in total number of minutes, distinct therapy types, and therapy intensity of acute therapy services across diagnoses. Our sample included patients receiving therapy while in acute care, and approximately 90% of all MS-DRG codes were observed, indicating that almost all conditions treated in hospitals receive some form of rehabilitation service. Consistent with other studies,^[12,15]^ we found that larger hospitals have higher therapy utilization, but given that we observed no consistent pattern between hospital size and number of distinct therapy types or therapy intensity, the main driver of this relationship remains unclear.

The 3 conditions that received the highest levels of therapy services were MJR, septicemia, and stroke. MJR had the lowest proportion of patients with comorbidities and complications (4.7%) and the lowest proportion with extended hospital stays (13.1%), but approximately 90% received high total therapy minutes (i.e., ≥120 minutes), high number of distinct therapy types (≥2 services), and high therapy intensity (≥30 minutes per day). Moreover, there was little variability between hospitals on all therapy metrics for patients with MJR. While patients with stroke received the next highest level of service provision, there was greater variability between hospitals. Approximately 80% of the patients with stroke had comorbidities and complications, and 28% had extended hospital stays; 65% received the highest total number of minutes and 75% received high therapy intensity. These findings are similar to earlier reports of MJR and stroke samples,^[12]^ which could suggest a potentially disproportionate use of therapy services where most patients with MJR (90%) received the highest levels of therapy minutes, therapy intensity, and distinct therapy types, even when these patients had the lowest levels of comorbidities and complications relative to all other conditions. However, it is also possible that these differences are attributable to the distinct clinical presentations among conditions (e.g., more MJRs are planned and consequences of stroke may be more variable).

Variability in service provision across hospitals and conditions is likely compounded by competing evidence from the literature employing different metrics of therapy provision (defined as either total minutes, types, or intensity). In this study, patients with increased number of minutes and types of therapy services had increased odds of an extended LOS and discharge to PAC, while patients with increased therapy intensity had decreased odds of an extended LOS and no difference in the odds of PAC discharge. Overall, these findings are consistent with recently published studies in a variety of settings and conditions; indicating that therapy intensity can influence LOS, while discharge destination is a more complex process that involves factors beyond the individuals’ health status.^[15,16]^ Nevertheless, the strength of these effects differ substantially between conditions. Because our metrics of therapy provision were crude in this exploratory analysis, we cannot evaluate the relative merit of 1 metric to another nor can we generalize these findings to other settings. However, the differential effects we observed using different but positively correlated metrics based on the same data highlight the importance of multidimensional assessments of service provision and the need for careful consideration of the therapy metrics used to identify best practices for provision of acute therapy services. Given the emergence of advanced payment models (e.g., bundled payments) that include care provided outside of acute care hospitalizations, the impact of operational definitions should be explored in the context of these new definitions of an episode of care.^[14,17]^

### 4.1 Limitations

Readers should note several limitations. First, we did not have access to clinical information at the patient level nor detailed information regarding other interventions that may influence participation in therapy services, discharge, or discharge destination. Disease severity was measured using MS-DRG codes which is only a proxy of severity. Caution is warranted in interpreting effects of therapy services, given that one's general state of health is not sufficient to accurately describe the processes associated with discharge destination. Moreover, while statistically valid in terms of direction and strength, ORs as measures of true relative risk are likely inflated when the rare-event assumption is not met. Caution is warranted when interpreting the magnitude of risk represented by ORs given that a substantial number of patients experienced an extended LOS.

To our knowledge, this is the first study to quantify provision of acute therapy services directly instead of through revenue codes. This study overcomes several limitations of previous studies and highlights how a variety of therapy metrics can produce seemingly paradoxical findings with respect to outcomes. Future studies comparing the relative merit of competing metrics are warranted to identify optimal measures of service provision, given their instrumental role in informing clinical practice guidelines.

## Acknowledgment

A preliminary version of this analysis was presented at the Combined Sections Meeting of the American Physical Therapy Association in 2017 in San Antonio, TX.

## Author contributions

**Conceptualization:** Carmen E. Capo-Lugo, Robert L. Askew, Matthew Boebel, Christine DeLeo, Anne Deutsch, Allen Heinemann.

**Data curation:** Carmen E. Capo-Lugo, Robert L. Askew, Matthew Boebel.

**Formal analysis:** Carmen E. Capo-Lugo, Robert L. Askew, Anne Deutsch, Allen Heinemann.

**Funding acquisition:** Carmen E. Capo-Lugo, Allen Heinemann.

**Investigation:** Carmen E. Capo-Lugo.

**Methodology:** Carmen E. Capo-Lugo, Robert L. Askew, Matthew Boebel, Anne Deutsch, Allen Heinemann.

**Project administration:** Carmen E. Capo-Lugo, Christine DeLeo, Allen Heinemann.

**Resources:** Carmen E. Capo-Lugo, Matthew Boebel, Allen Heinemann.

**Software:** Carmen E. Capo-Lugo, Robert L. Askew.

**Supervision:** Carmen E. Capo-Lugo, Christine DeLeo.

**Validation:** Carmen E. Capo-Lugo, Robert L. Askew.

**Visualization:** Carmen E. Capo-Lugo, Robert L. Askew.

**Writing – original draft:** Carmen E. Capo-Lugo, Robert L. Askew.

**Writing – review & editing:** Carmen E. Capo-Lugo, Robert L. Askew, Matthew Boebel, Christine DeLeo, Anne Deutsch, Allen Heinemann.
